# Supplementary material for: Step-wise evolution of azole resistance through copy number variation followed by KSR1 loss of heterozygosity in Candida albicans
Source: PLoS Pathog. 2024 Aug 30;20(8):e1012497. doi: 10.1371/journal.ppat.1012497 (PMC11392398; doi:10.1371/journal.ppat.1012497)
Supplement: S1 Fig — A flow chart showing the sphingolipid biosynthesis pathway in C. albicans, compiled from [32,34,37,87,99]. Chemical inhibitors are shown in red. (PDF) [file ppat.1012497.s004.pdf]

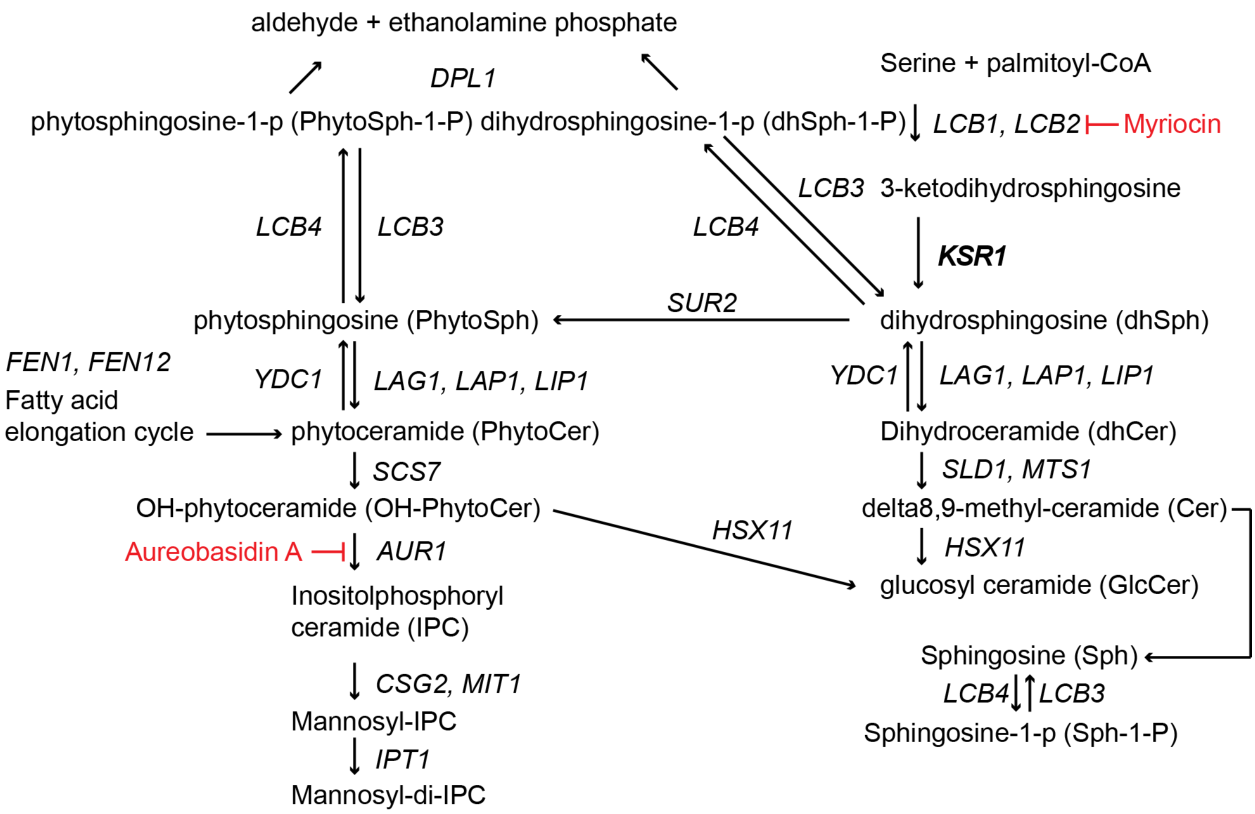

S1 Fig. The sphingolipid biosynthesis pathway in *C. albicans*. A flow chart showing the sphingolipid biosynthesis pathway in *C. albicans*, compiled from [32,34,37,87,99]. Chemical inhibitors are shown in red.

Additional Reference:

99. Ren J, Hannun YA. Metabolism and Roles of Sphingolipids in Yeast Saccharomyces cerevisiae. In: Geiger O, editor. Biogenesis of Fatty Acids, Lipids and Membranes. Cham: Springer International Publishing; 2017. pp. 1–21.
