# Supplementary material for: Step-wise evolution of azole resistance through copy number variation followed by KSR1 loss of heterozygosity in Candida albicans
Source: PLoS Pathog. 2024 Aug 30;20(8):e1012497. doi: 10.1371/journal.ppat.1012497 (PMC11392398; doi:10.1371/journal.ppat.1012497)
Supplement: S2 Fig — (A) Survival curves for mice infected with the progenitor strain SC5314 (red) or with the evolved P10 population (black, AMS4058) (Mantel-Cox log-rank test p-value = 0.0018). (B) Representative microscopy images show differences in hyphal formation between the progenitor strain SC5314 (left) and the evolved population (right) after incubation at 37°C for 2 h in rich media. (C) Read depth from whole genome sequencing normalized to average depth across the whole genome is shown for the evolved population, capped at an estimated 4 copies. Colors indicate changes in allele frequencies at heterozygous positions. Blue indicates an increase in the proportion of the “A” reference allele, while pink indicates an increase in the proportion of the “B” reference allele, signifying losses of heterozygosity. (D) Read depth from whole genome sequencing normalized to average depth across the whole genome is shown for the evolved population without a limit to the read depth for the full visualization of the CNV on Chr4. (C and D) Dots indicate positions of major repeat sequences. Blue dot indicates rDNA repeats. (PDF) [file ppat.1012497.s005.pdf]

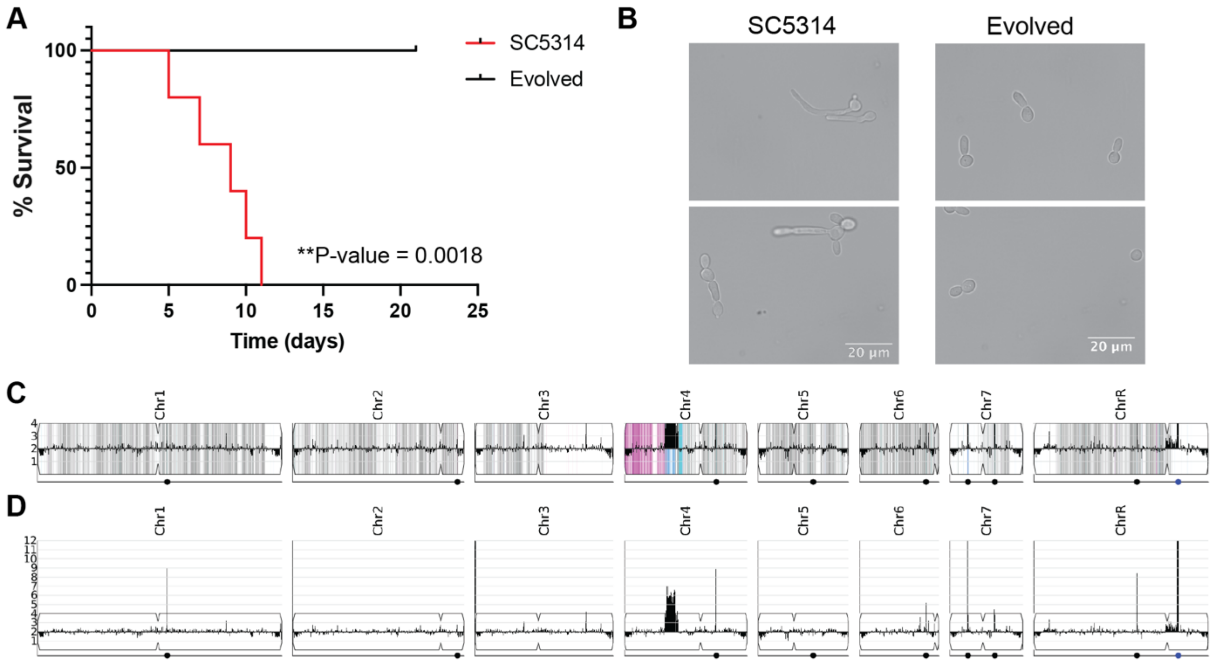


**S2 Fig. Properties of the evolved population.** (A) Survival curves for mice infected with the progenitor strain SC5314 (red) or with the evolved P10 population (black, AMS4058) (Mantel-Cox log-rank test p-value = 0.0018). (B) Representative microscopy images show differences in hyphal formation between the progenitor strain SC5314 (left) and the evolved population (right) after incubation at 37°C for 2 h in rich media. (C) Read depth from whole genome sequencing normalized to average depth across the whole genome is shown for the evolved population, capped at an estimated 4 copies. Colors indicate changes in allele frequencies at heterozygous positions. Blue indicates an increase in the proportion of the "A" reference allele, while pink indicates an increase in the proportion of the "B" reference allele, signifying losses of heterozygosity. (D) Read depth from whole genome sequencing normalized to average depth across the whole genome is shown for the evolved population without a limit to the read depth for the full visualization of the CNV on Chr4. (C and D) Dots indicate positions of major repeat sequences. Blue dot indicates rDNA repeats.
