# Supplementary material for: Step-wise evolution of azole resistance through copy number variation followed by KSR1 loss of heterozygosity in Candida albicans
Source: PLoS Pathog. 2024 Aug 30;20(8):e1012497. doi: 10.1371/journal.ppat.1012497 (PMC11392398; doi:10.1371/journal.ppat.1012497)
Supplement: S3 Fig — (A) Read depth from whole genome sequencing normalized to average depth across the whole genome is shown for 5 single colonies selected from passage 2 of the evolution experiment. Colors indicate changes in allele frequencies at heterozygous positions. Blue indicates an increase in the proportion of the “A” reference allele, while pink indicates an increase in the proportion of the “B” reference allele, signifying losses of heterozygosity. (B) OD600 values for liquid culture growth assays are plotted over time for the wild-type progenitor (black), and the four unique genotypes recovered from passage 2. Growth in rich media without drug, with 1 μg/mL or 256 μg/mL FLC are shown. Error bars are standard errors for three replicates. Tables on the right side indicate average MIC50 and SMG values for these genotypes. (C) Read depth plotted as in (A) for five single colonies selected from passage 3 of the evolution experiment. (D) OD values for liquid culture growth assays are plotted over time for the wild-type progenitor (black), and the two unique genotypes recovered from passage 3. Growth in rich media alone or with 1 μg/mL FLC or 256 μg/mL FLC are shown. Error bars are standard errors for three replicates. Tables on the right side indicate average MIC50 and SMG values for individual genotypes. (E) Read depth plotted as in (A) for the 5 single colonies selected from passage 4 of the evolution experiment. (PDF) [file ppat.1012497.s006.pdf]

A Five single colonies from passage 2

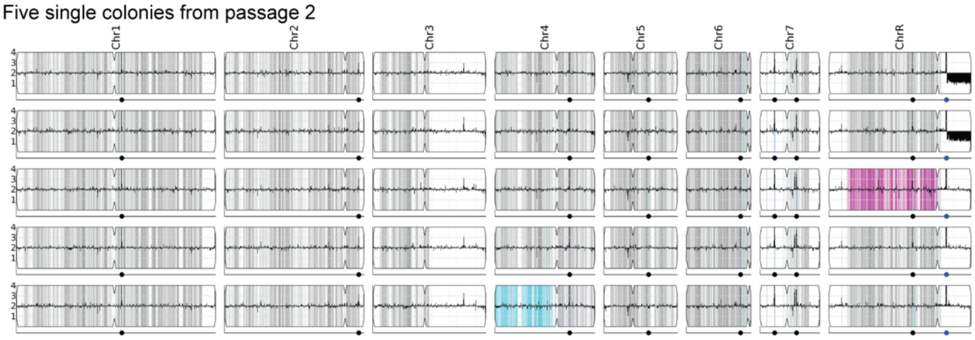

B

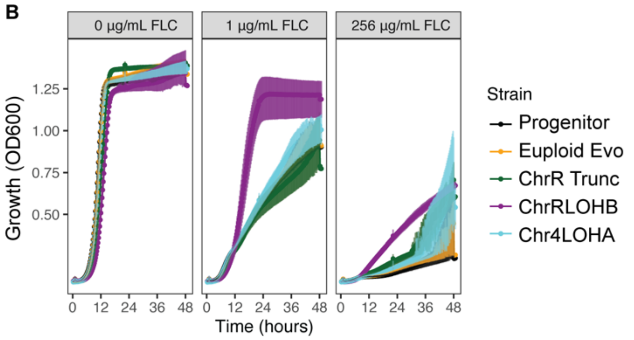

Strain	Microbroth dilution MIC	Microbroth dilution SMG
Progenitor	0.5	0.1
Euploid Evo	0.5	0.18
ChrR Trunc	0.5	0.27
ChrRLOHB	2	.078
Chr4LOHA	1	0.16

C Five single colonies from passage 3

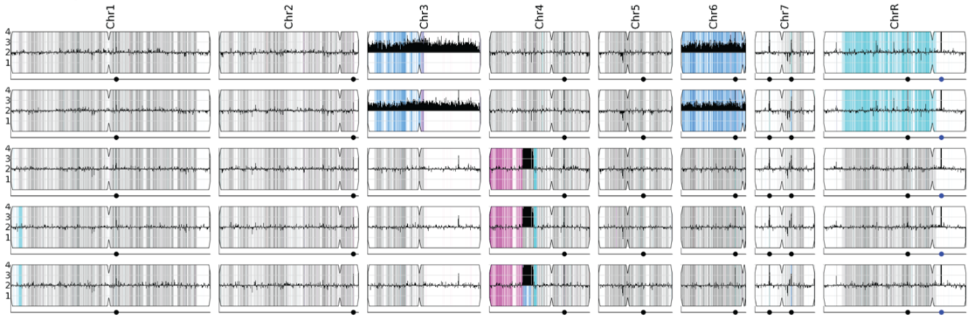

D

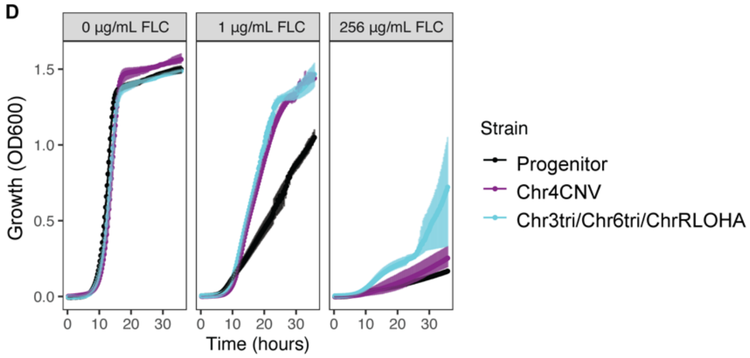

Strain	Microbroth dilution MIC	Microbroth dilution SMG
Progenitor	0.5	0.1
Chr4CNV	1	0.25
Chr3tri/Chr6tri/ChrRLOHA	1	0.3

E Five single colonies from passage 4 (P4.4 and P4.5 are the same colonies depicted in Figure 1G, I)

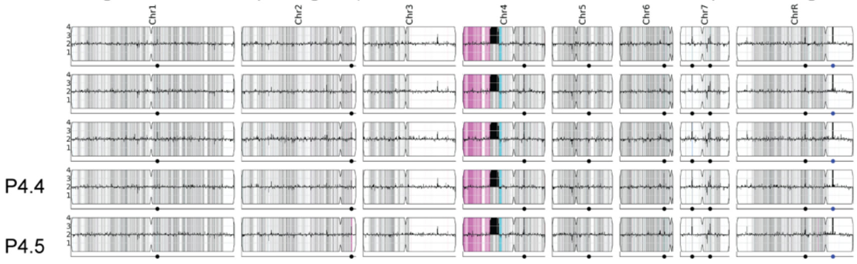

	Microbroth dilution MIC	Microbroth dilution SMG	
P4.4	1	0.27	P4.4: WT *KSR1*
P4.5	>256	NA	P4.5: *KSR1* LOH1

S3 Fig. Single colonies isolated from passages 2, 3, and 4. (A) Read depth from whole genome sequencing normalized to average depth across the whole genome is shown for 5 single colonies selected from passage 2 of the evolution experiment. Colors indicate changes in allele frequencies at heterozygous positions. Blue indicates an increase in the proportion of the “A” reference allele, while pink indicates an increase in the proportion of the “B” reference allele, signifying losses of heterozygosity. (B) OD_{600} values for liquid culture growth assays are plotted over time for the wild-type progenitor (black), and the four unique genotypes recovered from passage 2. Growth in rich media without drug, with 1 μg/mL or 256 μg/mL FLC are shown. Error bars are standard errors for three replicates. Tables on the right side indicate average MIC_{50} and SMG values for these genotypes. (C) Read depth plotted as in (A) for five single colonies selected from passage 3 of the evolution experiment. (D) OD values for liquid culture growth assays are plotted over time for the wild-type progenitor (black), and the two unique genotypes recovered from passage 3. Growth in rich media alone or with 1 μg/mL FLC or 256 μg/mL FLC are shown. Error bars are standard errors for three replicates. Tables on the right side indicate average MIC_{50} and SMG values for individual genotypes. (E) Read depth plotted as in (A) for the 5 single colonies selected from passage 4 of the evolution experiment.
