# Supplementary material for: Step-wise evolution of azole resistance through copy number variation followed by KSR1 loss of heterozygosity in Candida albicans
Source: PLoS Pathog. 2024 Aug 30;20(8):e1012497. doi: 10.1371/journal.ppat.1012497 (PMC11392398; doi:10.1371/journal.ppat.1012497)
Supplement: S4 Fig — (A) Estimated copy number, calculated as read depth normalized to the rest of the nuclear genome, is plotted for 500 bp windows across the left arm of chromosome 4. The position of the NCP1 gene is indicated in green. (B) RT-qPCR data show fold change in expression of NCP1 relative to the wild-type background strain. Fold changes are shown for the strain containing the tet-off NCP1 system in rich media without and with doxycycline and for the wild type strain (WT) and tet-off NCP1 strain grown in 1 μg/mL FLC with and without doxycycline (and supplemented with iron). Black line indicates a fold change of 1 (no change) relative to the wild type strain. Error bars are propagated standard error of three replicates. (C) RT-qPCR data show fold change in expression of NCP1 relative to the progenitor strain in the absence of FLC. Fold changes are shown for strain P3.3, which bears the Chr4 CNV in YPAD, and the wild type and P3.3 grown in 1 μg/mL FLC. (D) A heatmap showing relative growth after 24 hours for the progenitor strain and a strain containing the native NCP1 gene under the control of a tet-off promoter system in the absence (NCP1 on) and presence of doxycycline (NCP1 off), as well as supplemented with iron (+Fe) to counteract synergistic effects between doxycycline and fluconazole. Yellow lines indicate MIC50 values. (PDF) [file ppat.1012497.s007.pdf]

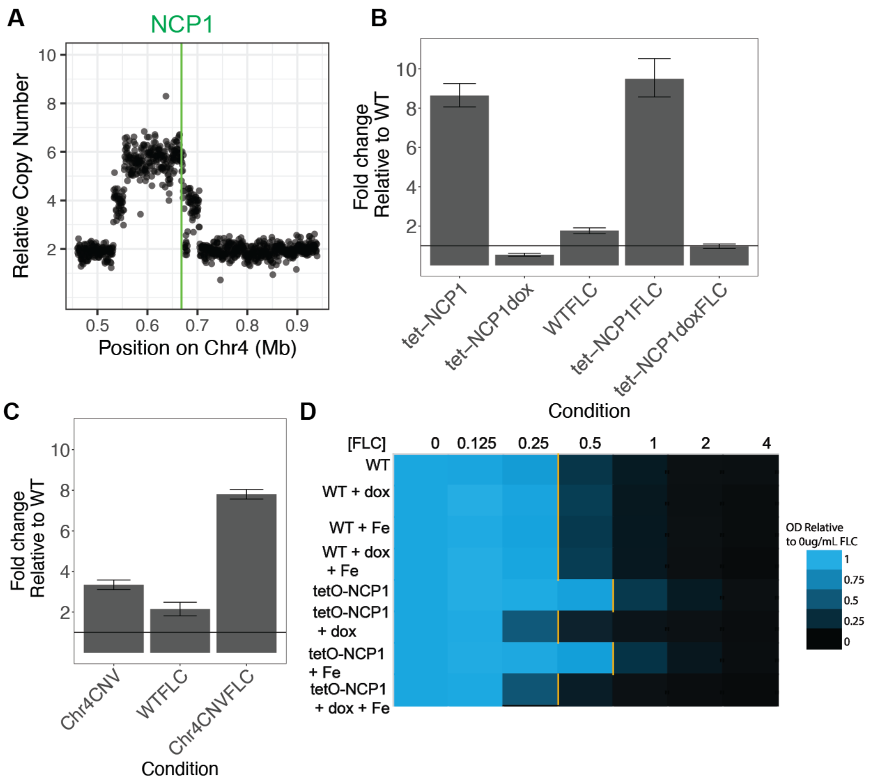

S4 Fig. Overexpression of *NCP1* leads to an increase in MIC$_{50}$. (A) Estimated copy number, calculated as read depth normalized to the rest of the nuclear genome, is plotted for 500 bp windows across the left arm of chromosome 4. The position of the *NCP1* gene is indicated in green. (B) RT-qPCR data show fold change in expression of *NCP1* relative to the wild-type background strain. Fold changes are shown for the strain containing the tet-off *NCP1* system in rich media without and with doxycycline and for the wild type strain (WT) and tet-off *NCP1* strain grown in 1 µg/mL FLC with and without doxycycline (and supplemented with iron). Black line indicates a fold change of 1 (no change) relative to the wild type strain. Error bars are propagated standard error of three replicates. (C) RT-qPCR data show fold change in expression of *NCP1* relative to the progenitor strain in the absence of FLC. Fold changes are shown for strain P3.3, which bears the Chr4 CNV in YPAD, and the wild type and P3.3 grown in 1 µg/mL FLC. (D) A heatmap showing relative growth after 24 hours for the progenitor strain and a strain containing the native *NCP1* gene under the control of a tet-off promoter system in the absence (*NCP1* on) and presence of doxycycline (*NCP1* off), as well as supplemented with iron (+Fe) to counteract synergistic effects between doxycycline and fluconazole. Yellow lines indicate MIC$_{50}$ values.
