# Supplementary material for: Step-wise evolution of azole resistance through copy number variation followed by KSR1 loss of heterozygosity in Candida albicans
Source: PLoS Pathog. 2024 Aug 30;20(8):e1012497. doi: 10.1371/journal.ppat.1012497 (PMC11392398; doi:10.1371/journal.ppat.1012497)
Supplement: S5 Fig — (A) OD600 values for liquid culture growth assays are plotted over time for the wild type progenitor (black), a strain engineered to contain a tet-off-NCP1 allele (light green) and a strain engineered to contain both the tet-off-NCP1 allele and the KSR1 LOH1 (dark green). Growth in rich media without drug, 1 μg/mL FLC, or 256 μg/mL FLC are shown. Error bars are standard error for three replicates. MIC50 and SMG values calculated at 24 and 48 hours are shown to the left (see Methods). (B) As in (A), OD values for liquid culture growth assays are plotted over time for the wild type progenitor (black), a strain engineered to contain a tet-off-NCP1 allele (light green), and the same strain grown in the presence of doxycycline (orange). Growth in rich media, 1 μg/mL FLC, and 256 μg/mL FLC are shown. (PDF) [file ppat.1012497.s008.pdf]

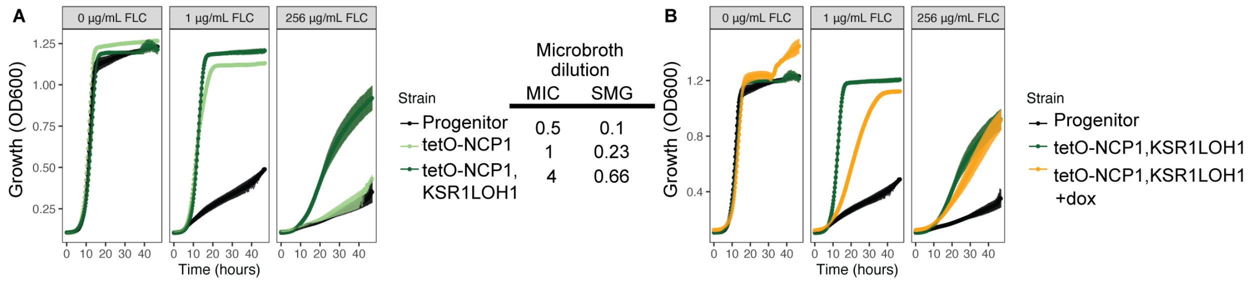

S5 Fig. Combined effect of *NCP1* overexpression and *KSR1* LOH1.

(A) OD_{600} values for liquid culture growth assays are plotted over time for the wild type progenitor (black), a strain engineered to contain a tet-off-NCP1 allele (light green) and a strain engineered to contain both the tet-off-NCP1 allele and the *KSR1* LOH1 (dark green). Growth in rich media without drug, 1 µg/mL FLC, or 256 µg/mL FLC are shown. Error bars are standard error for three replicates. MIC_{50} and SMG values calculated at 24 and 48 hours are shown to the left (see Methods). (B) As in (A), OD values for liquid culture growth assays are plotted over time for the wild type progenitor (black), a strain engineered to contain a tet-off-*NCP1* allele (light green), and the same strain grown in the presence of doxycycline (orange). Growth in rich media, 1 µg/mL FLC, and 256 µg/mL FLC are shown.
