# Supplementary material for: Step-wise evolution of azole resistance through copy number variation followed by KSR1 loss of heterozygosity in Candida albicans
Source: PLoS Pathog. 2024 Aug 30;20(8):e1012497. doi: 10.1371/journal.ppat.1012497 (PMC11392398; doi:10.1371/journal.ppat.1012497)
Supplement: S6 Fig — (A) A heatmap showing OD600 values relative to growth in rich media after 24 hours in a broth microdilution assay for engineered strains with homozygous or heterozygous deletions of KSR1. The engineered strain containing the same KSR1 genotype that evolved in KSR1 LOH1 is shown last for comparison. Yellow lines indicate the FLC MIC50. (B) Read depth from whole genome sequencing normalized to average depth across the whole genome is shown for 4 single colonies with complete deletions of KSR1. Colors indicate changes in allele frequencies at heterozygous positions. Blue indicates an increase in the proportion of the “A” reference allele, while pink indicates an increase in the proportion of the “B” reference allele, signifying losses of heterozygosity. (C) Read depth and SNV composition from integrated genomics viewer (IGV) are shown for one evolved isolate (Evolved P10) and engineered homozygous knockout mutants in SC5314 and in BWP17 backgrounds. All four full knockout mutants show losses of heterozygosity, indicated by full color SNVs, including orf19.6134 and PIF1 which are all homozygous for the reference “A” allele. KSR1 is flanked by the uncharacterized orf19.6123 and MRLP8. (PDF) [file ppat.1012497.s009.pdf]

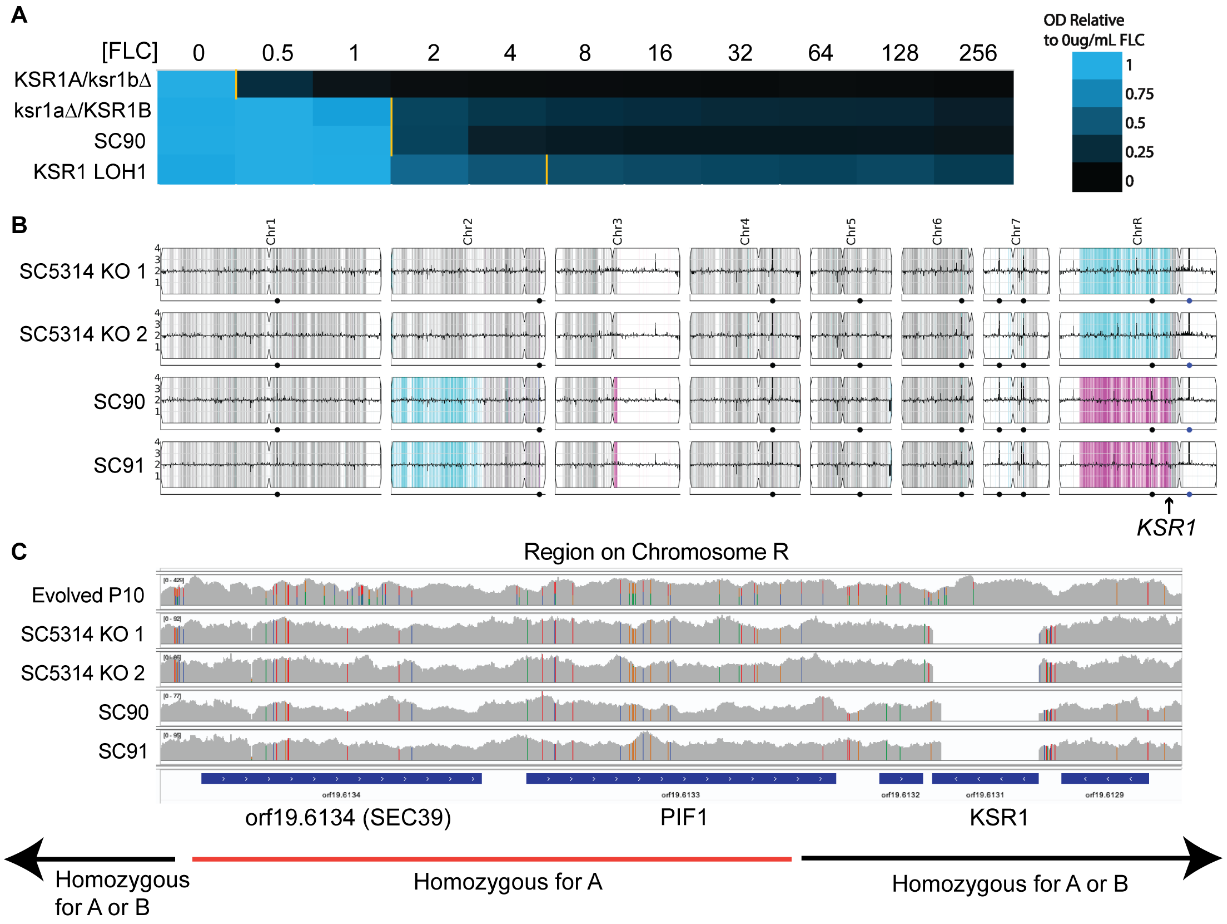

S6 Fig. Heterozygous and homozygous deletion mutants of *KSR1*

(A) A heatmap showing OD_{600} values relative to growth in rich media after 24 hours in a broth microdilution assay for engineered strains with homozygous or heterozygous deletions of *KSR1*. The engineered strain containing the same *KSR1* genotype that evolved in *KSR1* LOH1 is shown last for comparison. Yellow lines indicate the FLC MIC_{50}. (B) Read depth from whole genome sequencing normalized to average depth across the whole genome is shown for 4 single colonies with complete deletions of *KSR1.* Colors indicate changes in allele frequencies at heterozygous positions. Blue indicates an increase in the proportion of the "A" reference allele, while pink indicates an increase in the proportion of the "B" reference allele, signifying losses of heterozygosity. (C) Read depth and SNV composition from integrated genomics viewer (IGV) are shown for one evolved isolate (Evolved P10) and engineered homozygous knockout mutants in SC5314 and in BWP17 backgrounds. All four full knockout mutants show losses of heterozygosity, indicated by full color SNVs, including *orf19.6134* and *PIF1* which are all homozygous for the reference "A" allele. *KSR1* is flanked by the uncharacterized *orf19.6123* and *MRLP8*.
