# Supplementary material for: Step-wise evolution of azole resistance through copy number variation followed by KSR1 loss of heterozygosity in Candida albicans
Source: PLoS Pathog. 2024 Aug 30;20(8):e1012497. doi: 10.1371/journal.ppat.1012497 (PMC11392398; doi:10.1371/journal.ppat.1012497)
Supplement: S7 Fig — (A) RT-qPCR data for one biological replicate in technical triplicate is shown for expression of the gene CDR1 relative to the gene ACT1 for the KSR1 LOH1 engineered strain, the KSR1 LOH1 engineered strain growth in 1 μg/mL FLC, and the progenitor strains grown in 1 μg/mL FLC, all relative to the WT (progenitor) strain grown in rich media. Error bars are standard deviations for technical triplicate with propagation of error during delta-delta CT calculations. (B) Data for the first biological replicate, calculated as in (A) but normalized to the gene TEF1 rather than ACT1. (C) As in (A), RT-qPCR data for a second biological replicate in technical triplicate. (PDF) [file ppat.1012497.s010.pdf]

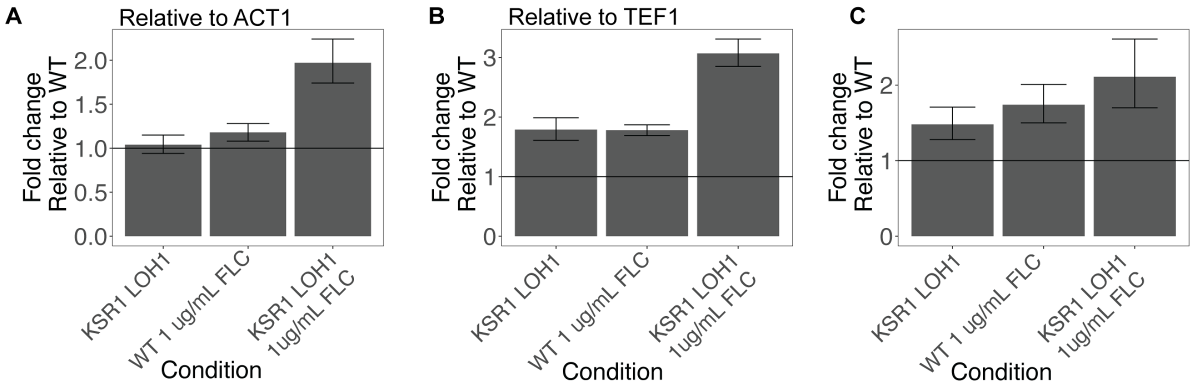

S7 Fig. RT-qPCR for *CDR1* in *KSR1* LOH1 strain. (A) RT-qPCR data for one biological replicate in technical triplicate is shown for expression of the gene *CDR1* relative to the gene *ACT1* for the *KSR1* LOH1 engineered strain, the *KSR1* LOH1 engineered strain growth in 1 μg/mL FLC, and the progenitor strains grown in 1 μg/mL FLC, all relative to the WT (progenitor) strain grown in rich media. Error bars are standard deviations for technical triplicate with propagation of error during delta-delta CT calculations. (B) Data for the first biological replicate, calculated as in (A) but normalized to the gene *TEF1* rather than *ACT1*. (C) As in (A), RT-qPCR data for a second biological replicate in technical triplicate.
