# Supplementary material for: Step-wise evolution of azole resistance through copy number variation followed by KSR1 loss of heterozygosity in Candida albicans
Source: PLoS Pathog. 2024 Aug 30;20(8):e1012497. doi: 10.1371/journal.ppat.1012497 (PMC11392398; doi:10.1371/journal.ppat.1012497)
Supplement: S8 Fig — Growth of each strain on RPMI agar plates with Amphotericin B e-test strips are shown for the wild type progenitor strain (top, AMS2401), and KSR1 mutant strains. E-strip readings are shown to the right of the strain collection number. (PDF) [file ppat.1012497.s011.pdf]

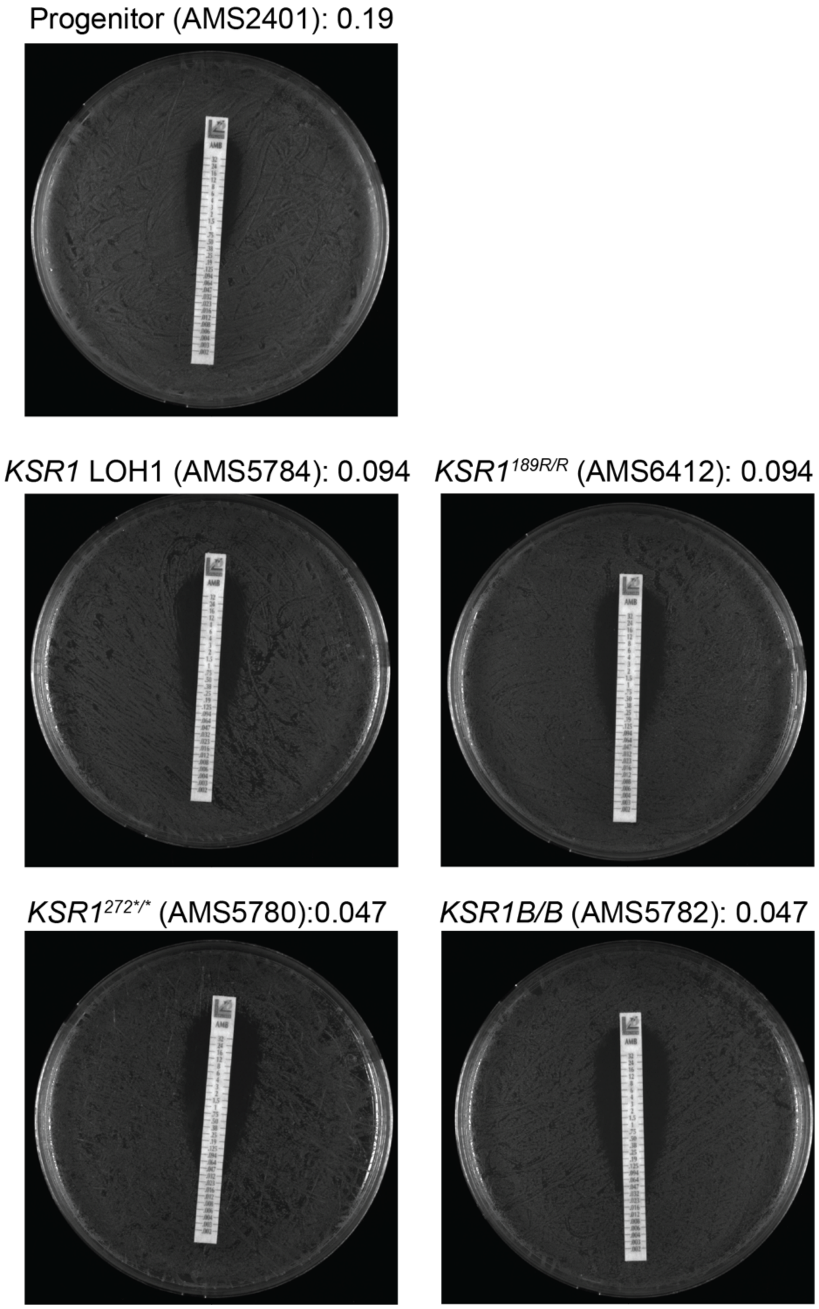

S8 Fig. *KSR1* LOH strains show increased sensitivity to Amphotericin B. Growth of each strain on RPMI agar plates with Amphotericin B e-test strips are shown for the wild type progenitor strain (top, AMS2401), and *KSR1* mutant strains. E-strip readings are shown to the right of the strain collection number.
