# Supplementary material for: Step-wise evolution of azole resistance through copy number variation followed by KSR1 loss of heterozygosity in Candida albicans
Source: PLoS Pathog. 2024 Aug 30;20(8):e1012497. doi: 10.1371/journal.ppat.1012497 (PMC11392398; doi:10.1371/journal.ppat.1012497)
Supplement: S9 Fig — (A) Read depth from whole genome sequencing normalized to average depth across the whole genome for three evolved strains that all contain LOH at the KSR1 locus and their SC5314 progenitor. Gray bars show heterozygous positions. (B) Microscopy of progenitor and evolved strains grown at 37°C for 2 hours shows differences in the initiation of hyphal growth. (C) Survival curves for G. mellonella show a reduction in virulence for all three evolved strains relative to the progenitor (Log-rank test, ** P < 0.01). (D) Median G. mellonella survival over 14 days demonstrates that treatment with FLC increases survival of the wild-type strain (gray) but does not increase survival for larvae infected with evolved strains LOH3 (green) and LOH4 (blue). (PDF) [file ppat.1012497.s012.pdf]

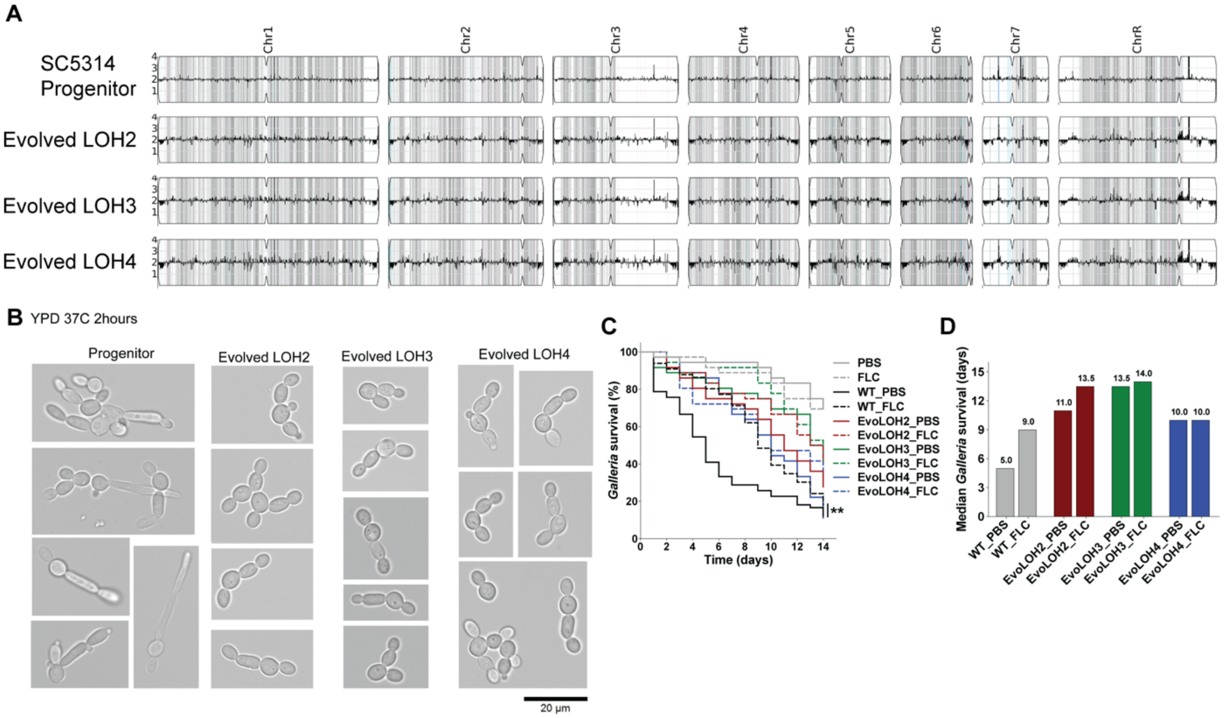

S9 Fig. Additional evolved strains with LOH at *KSR1*. (A) Read depth from whole genome sequencing normalized to average depth across the whole genome for three evolved strains that all contain LOH at the *KSR1* locus and their SC5314 progenitor. Gray bars show heterozygous positions. (B) Microscopy of progenitor and evolved strains grown at 37℃ for 2 hours shows differences in the initiation of hyphal growth. (C) Survival curves for *G. mellonella* show a reduction in virulence for all three evolved strains relative to the progenitor (Log-rank test, ** $P < 0.01$). (D) Median *G. mellonella* survival over 14 days demonstrates that treatment with FLC increases survival of the wild-type strain (gray) but does not increase survival for larvae infected with evolved strains LOH3 (green) and LOH4 (blue).
