# Supplementary material for: Step-wise evolution of azole resistance through copy number variation followed by KSR1 loss of heterozygosity in Candida albicans
Source: PLoS Pathog. 2024 Aug 30;20(8):e1012497. doi: 10.1371/journal.ppat.1012497 (PMC11392398; doi:10.1371/journal.ppat.1012497)
Supplement: S10 Fig — (A) Principal component analysis for RNA-sequencing data colored according to environmental condition. (B) Principal component analysis for RNA-sequencing data colored according to strain identity. (C) A heatmap showing normalized abundance from triplicate RNA-seq data for genes annotated as being related to sphingolipid biosynthesis. Rows are genes and columns are all samples, including the wild type progenitor, Evolved LOH2, Evolved LOH3, and Evolved LOH4 in the absence of FLC (YPD), 2 μg/mL FLC, or 64 μg/mL FLC. Row and column dendrograms from k-means clustering are shown on the top and left. (D) A heatmap shows the log2 fold changes from triplicate RNA-seq data for genes catalyzing the steps of the sphingolipid biosynthesis pathway (S1 Fig). Columns are strains Evolved LOH2, Evolved LOH3, and Evolved LOH4 relative to the wild type progenitor in 0 μg/mL FLC, and rows are genes annotated as part of the sphingolipid biosynthesis pathway or related to sphingolipid transport. Fold changes that are significantly different at an adjusted p-value of 0.1 are indicated with an asterisk. (E) As in (D), a heatmap shows log2 fold changes from triplicate RNA-seq data for genes annotated as ABC transporters. (PDF) [file ppat.1012497.s013.pdf]

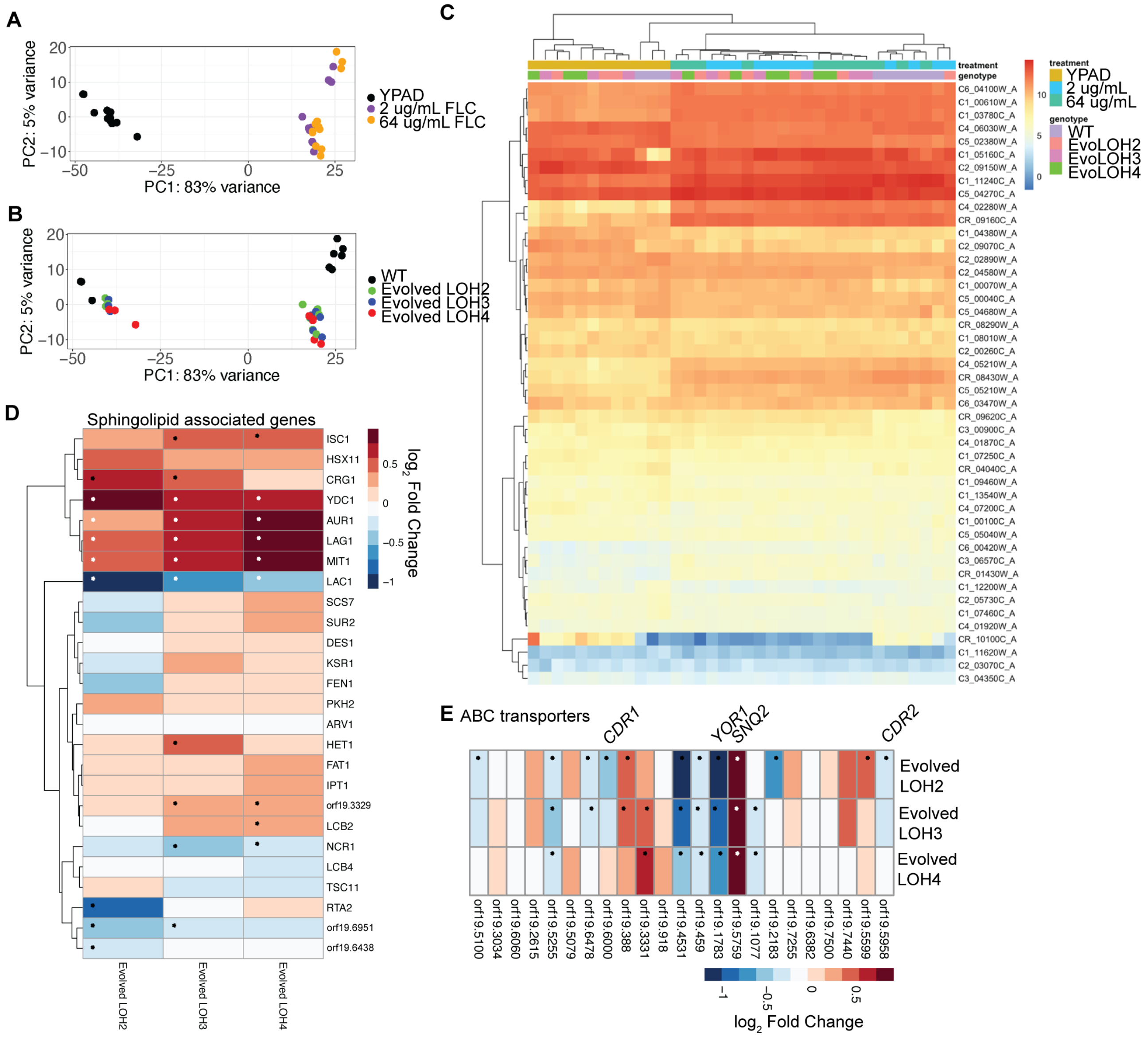

S10 Fig. RNA-sequencing analysis for Evolved LOH2, 3, and 4 isolates. (A) Principal component analysis for RNA-sequencing data colored according to environmental condition. (B) Principal component analysis for RNA-sequencing data colored according to strain identity. (C) A heatmap showing normalized abundance from triplicate RNA-seq data for genes annotated as being related to sphingolipid biosynthesis. Rows are genes and columns are all samples, including the wild type progenitor, Evolved LOH2, Evolved LOH3, and Evolved LOH4 in the absence of FLC (YPD), 2 µg/mL FLC, or 64 µg/mL FLC. Row and column dendrograms from k-means clustering are shown on the top and left. (D) A heatmap shows the $\log_2$ fold changes from triplicate RNA-seq data for genes catalyzing the steps of the sphingolipid biosynthesis pathway (Fig S1). Columns are strains Evolved LOH2, Evolved LOH3, and Evolved LOH4 relative to the wild type progenitor in 0 µg/mL FLC, and rows are genes annotated as part of the sphingolipid biosynthesis pathway or related to sphingolipid transport. Fold changes that are significantly different at an adjusted p-value of 0.1 are indicated with an asterisk. (E) As in (D), a

heatmap shows $\log_2$ fold changes from triplicate RNA-seq data for genes annotated as ABC transporters.
