# Supplementary material for: Step-wise evolution of azole resistance through copy number variation followed by KSR1 loss of heterozygosity in Candida albicans
Source: PLoS Pathog. 2024 Aug 30;20(8):e1012497. doi: 10.1371/journal.ppat.1012497 (PMC11392398; doi:10.1371/journal.ppat.1012497)
Supplement: S11 Fig — For each KSR1 mutant engineered strain, read depth is plotted on the y-axis according to genomic position on the x-axis. All strains shown are euploid (copy number of 2). In addition, grey bars show regions of heterozygosity, pink bars show regions of homozygosity of the “B” allele, and blue bars show homozygosity of the “A” allele. The tetO-NCP1, KSR1 LOH1 strain was engineered in the SN152 genetic background, that includes LOH on Chr2 and a small region of Chr3. Points below each diagram mark the position of major repeat sequences. (PDF) [file ppat.1012497.s014.pdf]

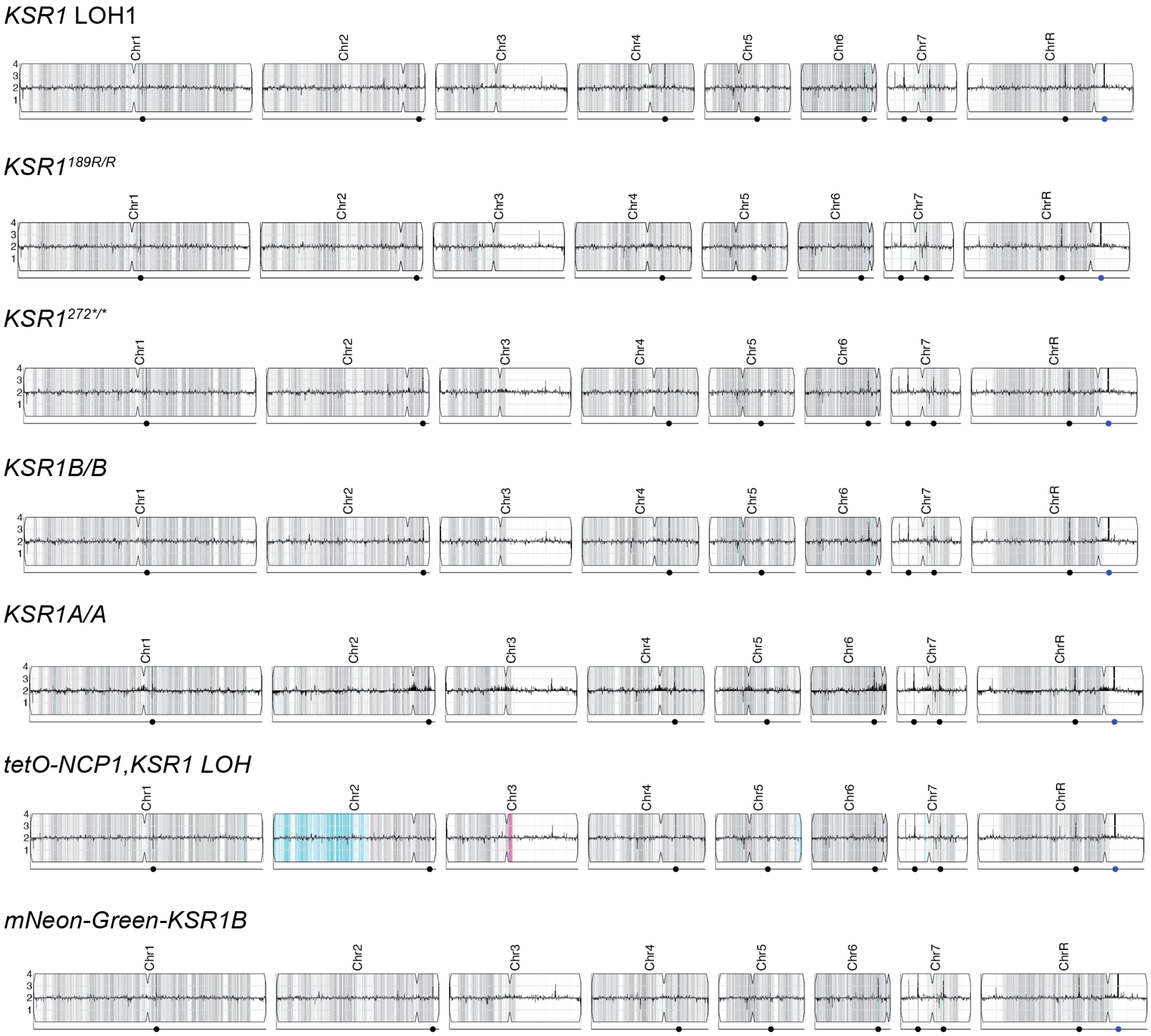

S11 Fig. Whole Genome Sequencing for all engineered *KSR1* strains. For each *KSR1* mutant engineered strain, read depth is plotted on the y-axis according to genomic position on the x-axis. All strains shown are euploid (copy number of 2). In addition, grey bars show regions of heterozygosity, pink bars show regions of homozygosity of the “B” allele, and blue bars show homozygosity of the “A” allele. The *tetO-NCP1, KSR1* LOH1 strain was engineered in the SN152 genetic background, that includes LOH on Chr2 and a small region of Chr3. Points below each diagram mark the position of major repeat sequences.
