# Supplementary material for: Step-wise evolution of azole resistance through copy number variation followed by KSR1 loss of heterozygosity in Candida albicans
Source: PLoS Pathog. 2024 Aug 30;20(8):e1012497. doi: 10.1371/journal.ppat.1012497 (PMC11392398; doi:10.1371/journal.ppat.1012497)
Supplement: S12 Fig — (A) Fluorescent microscopy (top rows) and DIC (subsequent rows) images for strains engineered to contain the KSR1 LOH1 or to be completely homozygous for KSR1 for either the A or B alleles. Cells were stained with the vacuolar dye FM4-64 after growth in YPD without drug (top) or after exposure to 128 μg/mL FLC (bottom). (B) Quantification of vacuolar area and integrated pixel intensity for fluorescence microscopy images shown in panel A, (see Methods). Points show an average of at least 200 cells and error bars represent standard errors of the mean (SEM). (C) As in A, fluorescence microscopy images of the additional three evolved strains with LOH affecting KSR1. (D) Quantification of fluorescence microscopy shown in panel (C). (B and D) Asterisks denote significant differences, using nonparametric t-tests, * P < 0.05, ** P < 0.01. (PDF) [file ppat.1012497.s015.pdf]

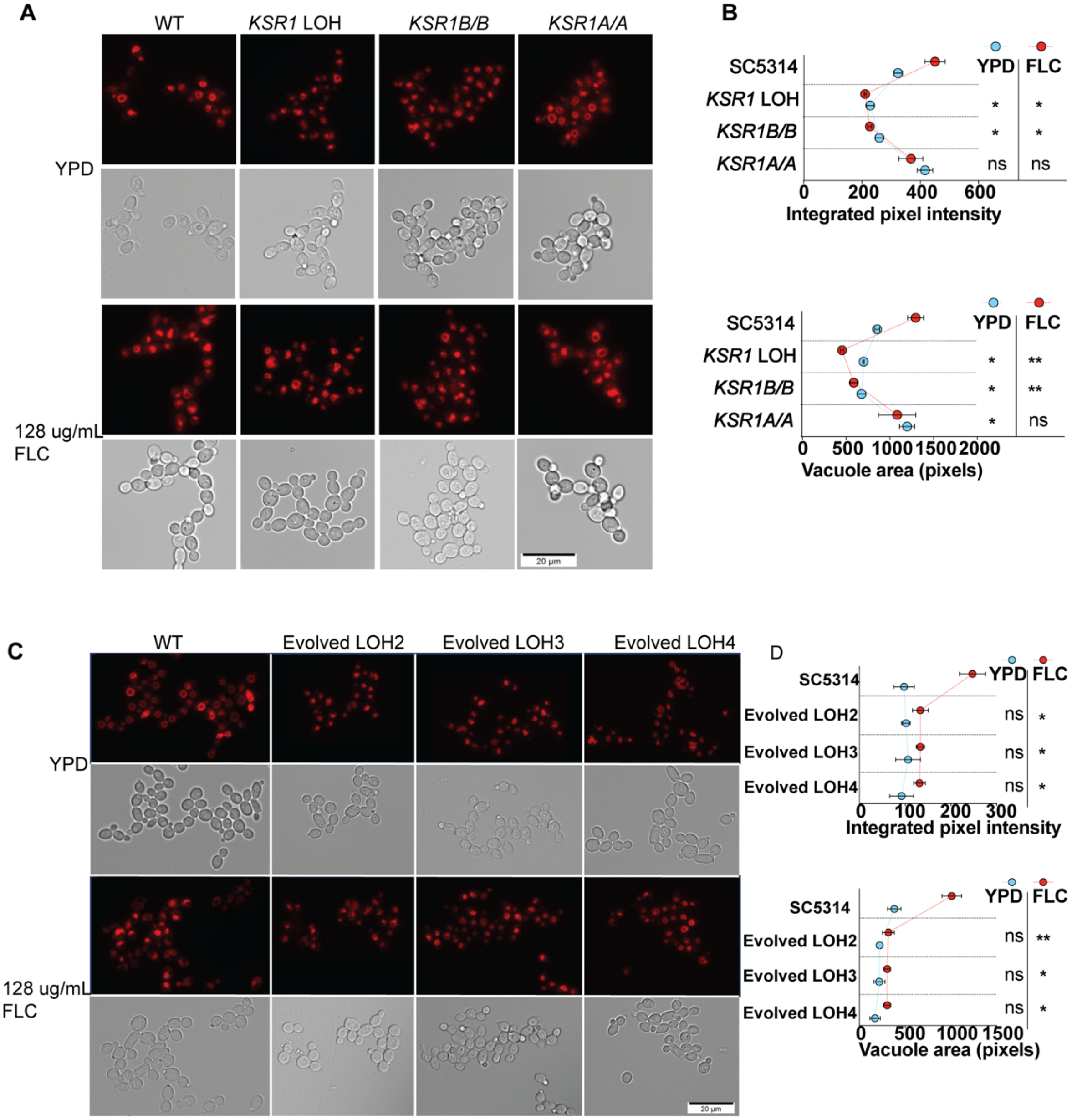

S12 Fig. Vacuolar phenotypes for evolved and engineered *KSR1* LOH mutants. (A) Fluorescent microscopy (top rows) and DIC (subsequent rows) images for strains engineered to contain the *KSR1* LOH1 or to be completely homozygous for *KSR1* for either the A or B alleles. Cells were stained with the vacuolar dye FM4-64 after growth in YPD without drug (top) or after exposure to 128 µg/mL FLC (bottom). (B) Quantification of vacuolar area and integrated pixel intensity for fluorescence microscopy images shown in panel A, (see Methods). Points show an average of at least 200 cells and error bars represent standard errors of the mean (SEM). (C) As in A, fluorescence microscopy images of the additional three evolved strains with LOH affecting *KSR1*. (D) Quantification of fluorescence microscopy shown in panel (C). (B and D) Asterisks denote significant differences, using nonparametric t-tests, * P < 0.05, ** P < 0.01.
