# Supplementary material for: Step-wise evolution of azole resistance through copy number variation followed by KSR1 loss of heterozygosity in Candida albicans
Source: PLoS Pathog. 2024 Aug 30;20(8):e1012497. doi: 10.1371/journal.ppat.1012497 (PMC11392398; doi:10.1371/journal.ppat.1012497)
Supplement: S13 Fig — (A) Fluorescent microscopy images for cells from strains engineered to contain the KSR1 LOH1 and to be completely homozygous for KSR1 for either the A or B allele. Cells were stained with the lipid droplet dye BODIPY after growth in YPD alone (top), after exposure to 128 μg/mL FLC (middle), or after exposure to 128 μg/mL myriocin (MYO, bottom). (B) Quantification of the number of lipid droplets, lipid size, and integrated pixel intensity for fluorescence microscopy images, a subset of which are shown in panel (A), (see Methods). Points are an average of at least 200 cells and error bars represent standard errors of the mean (SEM). (C) As in A, fluorescence microscopy images for the three additional evolved strains with LOH affecting KSR1. (D) Quantification of fluorescence microscopy, a subset of which is shown in panel (C). (B and D) Asterisks denote significant differences, using nonparametric t-tests, * P < 0.05, ** P < 0.01. (PDF) [file ppat.1012497.s016.pdf]

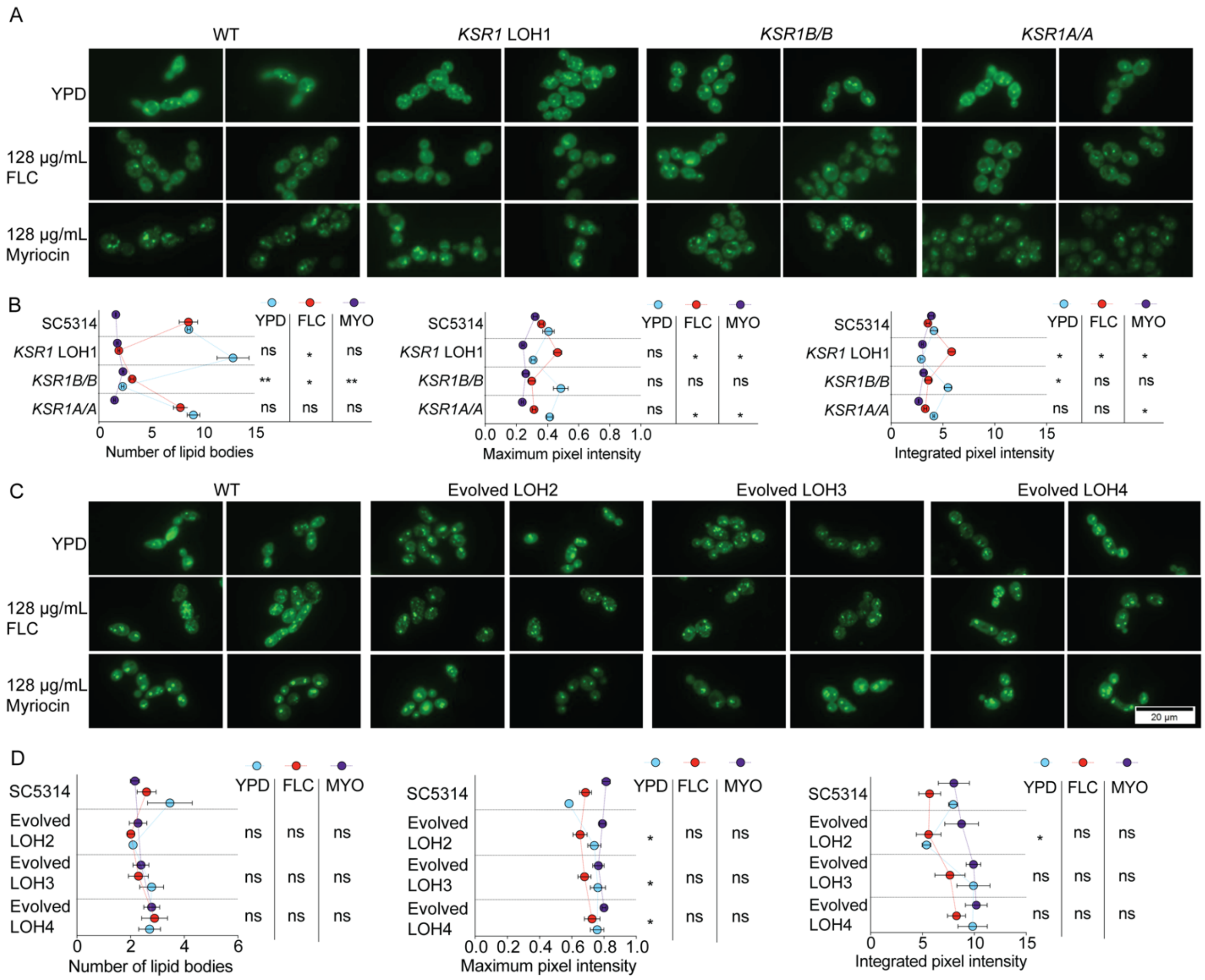

S13 Fig. BODIPY staining and microscopy for evolved and engineered *KSR1* mutants.
(A) Fluorescent microscopy images for cells from strains engineered to contain the *KSR1* LOH1 and to be completely homozygous for *KSR1* for either the A or B allele. Cells were stained with the lipid droplet dye BODIPY after growth in YPD alone (top), after exposure to 128 µg/mL FLC (middle), or after exposure to 128 µg/mL myriocin (MYO, bottom). (B) Quantification of the number of lipid droplets, lipid size, and integrated pixel intensity for fluorescence microscopy images, a subset of which are shown in panel (A), (see Methods). Points are an average of at least 200 cells and error bars represent standard errors of the mean (SEM). (C) As in A, fluorescence microscopy images for the three additional evolved strains with LOH affecting *KSR1*. (D) Quantification of fluorescence microscopy, a subset of which is shown in panel (C). (B and D) Asterisks denote significant differences, using nonparametric t-tests, * $P < 0.05$, ** $P < 0.01$.
