# Supplementary material for: Step-wise evolution of azole resistance through copy number variation followed by KSR1 loss of heterozygosity in Candida albicans
Source: PLoS Pathog. 2024 Aug 30;20(8):e1012497. doi: 10.1371/journal.ppat.1012497 (PMC11392398; doi:10.1371/journal.ppat.1012497)
Supplement: Attachment — Submitted filename: response_to_reviewers.pdf [file ppat.1012497.s017.pdf]

Response to Reviewers:

Part I - Summary
Please use this section to discuss strengths/weaknesses of study, novelty/significance, general execution and scholarship.

Reviewer #1: The manuscript by Petra Vande Zande et al. tried to dissect the mechanisms underlying the acquired fluconazole resistance in the human fungal pathogen Candida albicans during an in vitro evolution experiment. The authors performed elegant comparative genomic analyses and discovered two sequential events, large region CNV on Chr4 and a 711bp LOH of KSR1 gene on ChrR, that both conferred the FLC tolerance and resistance. They further employed genetic approaches to explore and validate the contribution of these genomic variants on FLC resistance/tolerance. The KSR1 LOH was linked to the sphingolipid biosynthesis and the activity of ABC transporter by biochemical assays. The authors also found that the homozygous locus of a nonsense mutation, KSR1 272*/*, influenced the fluconazole susceptibility via a different mechanism. While the genomic, genetic and biochemical data support the significance of these genomic variants in FLC resistance in C. albicans, this manuscript could be further strengthened if they focus more on the main phenotypes from the evolution experiment.

We thank the reviewer for their careful reading of this manuscript and insightful comments, and greatly appreciate the positive response to our genomic work. We believe the changes we have made do indeed bring more focus and strengthen the manuscript.

Reviewer #2: This study characterizes the genomic changes that occur upon long-term exposure to subinhibitory concentrations of fluconazole with the goal of understanding how genomic plasticity contributes to antifungal resistance. This study is well designed and in general the data support the conclusions. The results provide further insight to the mechanisms through which genomic changes can drive drug resistance in C. albicans while also highlighting a novel gene associated with azole resistance, KSR1. Overall this is nice work that contributes to an important aspect of fungal disease. It would benefit from some modifications to the language used to describe drug susceptibility and some revisions to address the following comments:

We appreciate the reviewer's kind words and suggestions for adjusting our usage language describing drug susceptibility. These changes as well as the additional experiments performed further strengthen the manuscript.

Reviewer #3: The manuscript presented by Zande et al, describes a novel perspective on the evolution of fluconazole tolerance/ resistance in C. albicans. Despite the fact that various studies have explored the impact of large- and small-scale genomic variations on azole tolerance/ resistance, the perspective on the stepwise evolutionary process among the azole adapted strains, especially at the single colony level in a large "clonal"

population, is lacking. Herein, authors employed whole-genomic sequencing on fluconazole adapted C. albicans isolates and by studying single colonies were able to identify novel and recurrent LOH potentially enhancing azole resistance, which increased the fluconazole MIC 512-fold when compared to the susceptible WT strain. By leveraging genetic engineering, lipidomic, RNAseq, microscopic, and phenotypic examination authors aimed at dissecting the role of LOH in KSR1 in fluconazole resistance. Additionally, authors identified that overexpression of NCP1 can also contribute to enhancing fluconazole MIC. Overall, the study is interesting.

We thank the reviewer for their close reading of the manuscript and interest in the study.

Part II – Major Issues: Key Experiments Required for Acceptance
Please use this section to detail the key new experiments or modifications of existing experiments that should be <u>absolutely</u> required to validate study conclusions.

Generally, there should be no more than 3 such required experiments or major modifications for a "Major Revision" recommendation. If more than 3 experiments are necessary to validate the study conclusions, then you are encouraged to recommend "Reject".

Reviewer #1: 1. Although the data related to the homozygosity of KSR1 272 */* nonsense mutation is quite interesting, this part of the data is quite distracted from the evolution experiment in this study. As the authors pointed out, none of the evolved strains with different patterns of KSR1 LOH include the homozygosity of this mutation. It is not part of the evolution events that the authors primarily aiming here. This part of data (the nonsense allele) could serve well in a separate study, such as to investigate the comprehensive function of Ksr1p in fluconazole resistance or in cell membrane integrity. The authors should reinforce the connection between this nonsense mutation and the acquired FLC resistance in the evolution experiment if they have other evidence not shown here, or could just remove this part of data from the manuscript.

We appreciate the reviewer’s point, and do not wish to distract the reader from the findings of the evolution experiments. However, we think that the precise breakpoints observed in all four evolution experiments, which all occur between the codon which codes for amino acid 189 and the early stop codon, are notable as they suggest that there was strong selection against complete homozygosity of the allele. This observation provides a direct link between the evolution experiment and the section describing the effects of the nonsense allele. We have emphasized this connection between the evolution experiment and the section pertaining to the nonsense mutation by changing the section header to “Recurrent and precise LOH breakpoint unlinks the effect of 189R/R from the masking effect of the nonsense codon” and modifying line 681 to read “The precision of the right LOH breakpoint between aa189 and aa272 in all four

evolved strains could indicate strong selective pressure against homozygosis of the nonsense codon at aa272."

2. On the other hand, the authors did not provide sufficient evidence to support the role of the homozygosity of KSR1 189R/R in FLC resistance. They only showed the fold change in MIC50 and SMG and the growth curve (Fig.3), and the sensitivity to SDS (Fig.4) of the KSR1 189R/R strain. If they claim that the LOH of this mutation is "a major driver of resistance" (line 460), they should provide more data showing the function of this mutation, such as the intracellular fluconazole concentration and the activity of the drug transporter (R6G fluorescence) of the KSR1189R/R strain, as they did with the KSR1 LOH1 strain in the Fig.2 B-D. Also, in the model cartoon presented in Fig.5C, if the authors suggested the KSR1 189R/R mutation affecting the sphingolipid composition, they need to perform lipid assays (Fig.2A) with the KSR1 189R/R strain and show the data profile resembles that of KSR1 LOH1.

As discussed in our response to the following comment, we reconstructed the *KSR1*$^{189R/R}$ mutant in a clean genetic background without any aneuploidy or additional loss of heterozygosity. With this new mutant, we re-collected the data we provided for the *KSR1*$^{189R/R}$ mutant originally, including MIC_{50}, SMG, and growth curves. We also added new data in this clean strain including sensitivity to SDS, efflux of rhodamine 6G, and intracellular fluconazole, which allow us to confidently ascribe the effects to *KSR1*$^{189R/R}$ alone. Notably, all of this phenotypic data has a strong similarity to the phenotypes measured for the engineered *KSR1* LOH1 strain (see **updated Figures 3 and 4**). Taken together, these data strengthen the argument *KSR1*$^{189R/R}$ alone is a major driver of the phenotypes observed in the *KSR1* LOH1 strain. Because of the difficulty of the lipidomics assays performed on the previous set of strains, we did not collect additional lipidomics data for this *KSR1*$^{189R/R}$ mutant; thus, we adjusted the model presented in Fig.5C, as well as the figure legend, by placing the *KSR1* LOH1 strain in the center of the fitness landscape with *KSR1*$^{189R/R}$ as the likely feature of the LOH that is most important.

3. According to Fig. S11, there is a large LOH segment of the ChrR in the KSR1 189R/R strain? If so, how would the authors conclude the increased FLC resistance of the KSR1 189R/R strain is driven by the homozygosity of the 189R/R locus? Similarly, although all carried the homozygous 189 R/R locus of KSR1, the three evolved strains LOH2/3/4 only showed modest increase of fluconazole resistance (Fig.3B). The influence of this KSR1 189R/R locus on the FLC resistance seems quite dependent on the other genomic variants. I strongly recommend the authors to construct a new point mutation strain and re-examine the phenotypes with a "cleaner" genome background, or at least state the limitation of the current strain in the result and discussion clearly.

As noted above, we constructed new mutants that are homozygous for arginine at *KSR1* amino acid 189 (*KSR1*$^{189R/R}$) and found a mutant that lacked any additional losses of heterozygosity, SNPs, or CNVs. We recollected all data for the *KSR1*$^{189R/R}$

strain using this new clean mutant and included the new panels in **Figures 3 and 4**. The whole genome sequencing depth and heterozygosity plot for this new strain is now found in **Supplementary Figure 11**.

Reviewer #2: 1. The introduction is very long; lines 91-133 could be shortened to remove excess details.

We thank the reviewer for this feedback and have abbreviated lines 91-133 (now lines 103-109), summarizing the results of several sphingolipid biosynthesis pathway studies at line 104: “In general, mutations or chemical inhibitors that disrupt steps in the sphingolipid biosynthesis pathway cause increased susceptibility or act synergistically with azoles [32-38].”

2. This manuscript uses loose or incorrect terminology to describe resistance/susceptibility.
Line 82 “Resistance is measured in the clinic as…” This is an incorrect definition of clinical resistance. Resistance in the clinic is defined as the ability of an organism to grow above defined clinical breakpoints. Though resistance is also commonly used to describe a strain that is less susceptible to a compound than its parent strain, this is not the clinical definition. In addition to correcting the clinical definition, the authors should explicitly state their definition of resistance used in this work.

We thank the reviewer for pointing this out and have adjusted the use of the terminology accordingly. (Lines 84 - 85 now read “Changes in drug susceptibility that result in clinical resistance, or the ability to grow above a clinical breakpoint, are measured as changes in MIC_{50}, the drug concentration that reduces growth by 50% or more at 24 h (Minimum Inhibitory Concentration, MIC_{50})”). In addition, we changed the usage of ‘resistance’ throughout the manuscript to ‘decreased susceptibility’ to better align with the clinical usage of these terms.

3. Similarly, throughout the manuscript the authors often say that strains are “more resistant” or that “CNV increase resistance by 500 fold.” Resistance itself is not a spectrum, strains are either resistant or they are not. The correct terminology to use here would be to say that strains are “less susceptible” or “500-fold less susceptible” than parent strains.

Once again, we thank the reviewer for this clarification and have adjusted the language accordingly, referring to the changes in MIC_{50} observed as decreased susceptibility rather than increased resistance.

4. Using MIC assays to determine whether strains are more or less susceptible to drug treatment is appropriate, however the authors have reported differences in MIC that fall within the error of this assay. Since it is a two-fold dilution series, a two-fold difference is considered within the error of the assay and does not represent a meaningful difference.

With two-fold dilutions, the limit of detection for a meaningful differences is a four-fold difference. Therefore at least a 4 fold change in MIC should be used as the threshold for resistance.

We thank the reviewer for raising this point. By changing the use of the term ‘resistance’ to ‘decreased susceptibility’,the 2-fold changes in MIC_{50} are no longer termed increases in resistance. In addition, for strains with 2-fold changes in susceptibility, we provide growth curve assay data with significant differences in growth rates and AUC measures at multiple drug concentrations. These assays better demonstrate the significant differences between the strains for their ability to grow in the presence of drug.

5. The conclusion that sphingolipids and azole resistance are mediated by efflux pumps is tenuous and the authors put too much stock into this as the mechanism of resistance in the KSR1 strains. Though it has been fairly well-accepted that resistance to azoles is mediated by the active efflux of azoles by CDR1 and CDR2, the data supporting this are also tenuous. The presence and absence of pumps in the membrane have a larger role in membrane homeostasis than just pumping out drugs/azoles. This is demonstrated by the observation that CDR1 or CDR2 null mutants have altered ergosterol profiles compared to wild type in the absence of fluconazole (PMID: 24435642). Moreover, these deletion strains are less susceptible to amphotericin while strains bearing overexpression of pumps are more susceptible to amphotericin, demonstrating that pumps are doing something else that is not related to pumping out fluconazole (and likely related to the sterol/lipid content or organization of the membrane) to modulate susceptibility to membrane targeting drugs. Other membrane targeting compounds such as fluphenazine also drive extremely high expression of these pumps but do not appear to be subject to efflux by the pump themselves, further supporting the role of pumps in maintaining membrane homeostasis. Even more compelling evidence is that fluphenazine and fluconazole are antagonistic in C. albicans (as one would expect based on strong induction of CDR1 by fluphenazine), however in C. glabrata where CDR1 has been shown to efflux rhodamine and thought to contribute to azole resistance, treatment of C. glabrata with fluphenazine induces CDR1 strongly, but this does not result in antagonism with fluconazole, disconnecting the expression of pumps from azole resistance under these conditions (PMID: 37702508). It is possible that pumps are actively removing fluconazole from cells and this is driving the susceptibility phenotypes. However it is probably more likely that the activity of the pumps in the evolved strain is modulating the membrane in response to changes in sphingolipids and these changes alter the susceptibility to fluconazole.

We thank the reviewer for these thoughtful comments and references to relevant literature, which we incorporated in the main text. We agree that the mechanism linking changes in sphingolipid levels and drug resistance is likely complex and due to more than the activation of efflux pumps simply reducing intracellular fluconazole levels and restoring ergosterol levels in mutant *KSR1* strains. We now clarify and emphasize the fact that much is still unknown about the interplay between drug efflux pumps, membrane homeostasis, and azole (and polyene) drug sensitivity. Lines 74-77 in the introduction now read “However, the most prevalent resistance mechanisms affect the

expression or activity of drug efflux pumps [1,3,18–21], which affect intracellular drug levels and influence membrane homeostasis in ways that affect drug susceptibility [22,23].” In addition, the results section has been heavily modified in light of new experiments described in response to the following comment below.

Deletion of the pumps in the KSR1 allele backgrounds would be more compelling evidence for the role of efflux in these cells, as cdr1 mutants are only moderately more susceptible to fluconazole than wild type C. albicans. If the susceptibility in these strains returns this strain to near WT levels of fluconazole susceptibility then the argument for efflux as the mechanism of resistance would be substantiated however these studies could be confounded by changes in the membrane that occur upon loss of CDR (or compensation by other pumps). Profiling the sterols and sphingolipids in all of these strains might help to build models where certain membrane states favor higher pump expression/activity. Even just profiling the sterols in addition to sphingolipids in the strains presented in this manuscript would at least determine whether sterol content differs or not, and if so, this would likely be a major player in the susceptibility to azoles.

We agree that deletion of efflux pumps is likely to have many pleiotropic phenotypic effects that will be difficult to interpret. In addition, while we agree that the efflux of FLC itself may not be the main mechanism of reduced drug susceptibility, we think that the activity of the drug efflux pumps in membrane homeostasis is likely to be important for the membrane composition changes that accompany reduced susceptibility. Therefore, as suggested, we profiled sterols and sphingolipids in the wild type and *KSR1* LOH1 strains to identify how sterol content differs in the presence and absence of fluconazole. These new experiments demonstrate that ergosterol levels are indeed elevated in the *KSR1* LOH1 strain in the absence of fluconazole (**new Figure 2 panels E-F**), similar to other strains with overexpression of efflux pumps *CDR1/CDR2* (Ren et al., 2014). In addition the *KSR1* LOH1 strain shows increased amphotericin B sensitivity (**new Figure S8**). In the presence of fluconazole, ergosterol content decreases to a similar level in both the wild type and *KSR1* LOH1 strains, demonstrating that increased efflux pump activity in the *KSR1* LOH1 strains does not restore Erg11p function and increased ergosterol content. Rather, the abundance of the toxic dienol 14α-methylergosta-8,24(28)-dien-3β,6α-diol is significantly reduced in the *KSR1* LOH1 strain relative to the wild type strain. This experiment provides support for the conclusion that changes to membrane homeostasis due to a reduced abundance of sphingolipid species and related to changes in efflux pump activity result in decreased azole susceptibility. These new findings are described in lines 537-555.

6. Similarly, the data presented for Cy5-FLU accumulation of cells could be the result of altered uptake due to changes in membrane permeability and function rather than drug efflux. It appears the authors did perform positive controls with beauvericin, it might be helpful to show that with beauvericin treatment, the KSR1 LOH strain accumulates Cy5-FLU to the same extent as the WT after 4 hours to support efflux rather than import defects.

In response to this comment, we collected additional data on the effect of beauvericin on intracellular fluconazole concentrations in the ksr1 mutant strains (in **new panel Figure 2D**). Importantly, preventing drug efflux activity of ABC transporters by beauvericin does not restore intracellular fluconazole levels to wild type levels, suggesting that there are also differences in uptake, activity by other efflux pumps, or other differences in membrane permeability.

7. This manuscript contains a LOT of data. Most of the supporting data is essential for supporting the conclusions of the paper or strain confirmation however the ceramide data did not add much in my opinion. The authors had previously shown in Figure 1G that the progenitor with Chr 4 CNV (p4.4) does not have a fitness cost, so it is not clear why the LOH event would be suspected of masking a fitness cost due to Chr 4 CNV.

We appreciate that this section in particular may be too distracting from the main points of the paper, and thus we removed it from the manuscript.

Reviewer #3: 1. Please ensure that the addition of doxycycline concentration used in the tet-off system does not have any impact on the growth of C. albicans in the absence and presence of fluconazole. Perhaps using a strong constitutive promoter, such as TEF1 promoter, would eliminate the concern of doxy impact on C. albicans growth. Along the same line, does the expression of baseline NPC1 in the absence of doxy matches that of the desired evolved strain?

We have included the appropriate controls to ensure that the addition of doxycycline is not affecting growth of *C. albicans*, either in the presence or absence of drug (**S4 Fig D**). Growth in doxycycline without fluconazole was comparable to growth in the absence of dox (WT OD = 1.00, WT + dox OD = 1.05). In addition, we added new data showing that strain P3.3, which contains the Chr4 CNV, overexpresses *NCP1* ~8-fold in the presence of fluconazole, which matches quite closely the 8-fold increased level of overexpression of the tetO system (See **updated S4 Fig C**).

2. Is it possible that the stepwise evolution is a misinterpretation of identified genotypes given that authors have explored only a limited number of single colonies in a population (5 colonies)? It could be possible that the genetic changes identified in P3 have occurred in P2, but at a lower frequency and they were not detected since authors investigated only 5 colonies (which is understandable given the limitations).

We agree that we did not exhaustively identify all genotypes that are present in the P2 and P3 populations. However, we clearly observe that the Chr4 CNV preceded the *KSR1* LOH, based on the presence of single colonies that contain the Chr4 CNV but not the *KSR1* LOH, and single colonies containing both mutations in later passages. In addition, because these cells are reproducing asexually, there is no opportunity for the LOH to arise in a different background and later introgress into the Chr4 CNV background. For these reasons, we are confident that the Chr4 CNV arose first, and the *KSR1* LOH occurred second in the same genetic background in this lineage.

3. Why authors studied the RNAseq 24hrs post-treatment? Similarly, why lipidomic experiments were conducted 48hrs post-treatment? This could result in inconsistency, complicate the analysis, and finally preclude authors from making potential links between RNAseq and lipidomic findings. For instance, measurement of intracellular level of fluconazole and efflux using Cy5-fluconazole and R6G at 4hrs post-infection is more reasonable and consistently shows the higher expression of an ATP-dependent efflux pump, potentially Cdr1, in pumping fluconazole outside of the cell.

The differences in collection procedures for RNAseq and lipidomics were constrained by the nature of the assays and also resulted from this collaboration between separate laboratories that performed the two assays independently before the initiation of the collaboration. For this reason, we were careful not to make direct comparisons between these datasets; rather we use both data types to independently support the hypotheses.

4. Along the previous comment, authors are recommended to individually delete CDR1, MDR1, SNQ2 in various KSR1 mutants and assess the fluconazole MIC and tolerance, intracellular fluconazole-Cy5, and R6G. This will at least elucidate which of those efflux pumps are the main driver of azole resistance/ tolerance in KSR1 variants conferring fluconazole resistance.

While we appreciate the suggestion, we do not think that deletion of each of these pumps individually will yield easily interpretable results for multiple reasons. First, it is very possible that the phenotypes we see are due to a combined effect of multiple pumps. Second, as suggested by another reviewer (Reviewer #2, comment 5), we edited the manuscript to better emphasize that fact that the phenotypes of intracellular FLC-Cy5, R6G efflux, and drug susceptibility are likely associated with structural changes in the membrane, and deleting individual transporters will likely have pleiotropic effects that will make interpretation difficult. We also collected extensive new sterol data showing: 1) in the absence of FLC, ergosterol levels are significantly higher in the *KSR1* LOH1 strain than in the progenitor, and 2) in the presence of FLC, ergosterol is not different between these two strains, but the toxic dienol (14α-methylergosta-8,24(28)-dien-3β,6α-diol) is significantly lower in the *KSR1* LOH1 strain than in the progenitor (**new Fig 2E,F**). Therefore, we adjusted our model to indicate that changes in membrane homeostasis, related to changes in efflux pump activity, reduce the susceptibility of the *KSR1* LOH1 strain to FLC.

5. The fact that KSR1 mutants have a higher MIC could be due to the excessive level of sphingolipids, rather than impacting the proper localization of efflux pumps in the membrane. Given that efflux pumps have numerous substrates and can be activated by multitude of compounds/ metabolic intermediates impacting yeast cell survival, the elevation of the efflux pumps could be an adaptive strategy to pump those toxic intermediates outside and therefore enhance the fitness, which coincidently confers fluconazole resistance?

We agree that changes in the sphingolipid composition (specifically, decreased sphingolipids in *KSR1* mutant strains) alter membrane homeostasis and potentially the activity of efflux pumps, and this altered membrane state reduces the cell's susceptibility to fluconazole. We collected new data showing that the level of ergosterol is elevated in *KSR1* LOH1 strain relative to the progenitor in the absence of fluconazole (**new Fig 2E**). Yet, ergosterol levels are depleted to a similar level in the presence of fluconazole in the *KSR1* LOH1 and progenitor strains. Rather, the presence of the toxic dienol (14α-methylergosta-8,24(28)-dien-3β,6α-diol), which has been shown to inhibit growth (Kelly et al, 1997) is reduced in the *KSR1* LOH1 strain relative to the progenitor (**new Fig 2F**). Therefore, we conclude that changes in membrane homeostasis, that are likely related to changes in drug efflux pump activity, result in lower sensitivity to fluconazole despite similar inhibition of Erg11p activity and ergosterol production.

6. Along the same line, KSR1 LOH showed an elevated level of Sph, dhSph-1P, and PhytoSph-1P. Therefore, it would be interesting to examine the impact of addition of each intermediate individually on C. albicans growth in the absence and presence of fluconazole, fluconazole MIC and tolerance levels, and intracellular fluconazole and efflux of R6G, and filamentation.

We agree this is an interesting question. The effect of adding dhSph-1-P and PhytoSph-1-P to a *C. albicans* reference strain has been previously assayed and found to indeed decrease sensitivity to the azole miconazole (VandenBosch et al, 2012). Given the amount of data in this paper already, we consider these questions beyond the scope of the manuscript and referenced this previous work in this section at lines 475-477.

7. CNVs have been shown to be very plastic and rapidly revert back to WT once passaged in the absence of fluconazole. Can the same thing be observed for KSR1 LOH?

Because it is not possible to restore heterozygosity once it is lost, we do not observe reversion of the *KSR1* LOH. CNVs are indeed very plastic and frequently revert back to wild type. However, continued passaging in the absence of fluconazole did not result in reversion of the Chr4 CNV for this lineage.

8. Can authors assess the prevalence of KSR1 LOH using the publicly available C. albicans whole-genome sequence data, especially LOH on the residue of 189?

We have included a new analysis examining the presence of both residues of interest in the *KSR1B* allele (arginine at 189, and an early stop codon at 272) across a set of 270 publicly available *Candida albicans* whole genome sequences (**new Table S3**). We found that these residues are restricted to SC5314-descended isolates. Furthermore, we do not observe homozygosis of these residues in sequenced isolates derived from SC5314 (apart from those described in this manuscript). We have included this new analysis in the discussion section at lines 878-880.

Part III – Minor Issues: Editorial and Data Presentation Modifications
Please use this section for editorial suggestions as well as relatively minor modifications of existing data that would enhance clarity.

Reviewer #1: 4. Is LOH1 the only different locus between the genomes of isolate P4.4 and P4.5? Could the difference of FLC resistance between the two isolates resulted from loci other than the Chr4 CNV and LOH1?

We did look for other variants called between these isolates using the same GATK variant calling pipeline used on the evolved isolates, and did not identify any additional copy number changes, losses of heterozygosity, or single nucleotide polymorphisms that differed between the P4.4 and P4.5 isolates. To the best of our knowledge, the LOH is the only genetic difference.

Also, as the authors showed, the combination of NCP1 overexpression and KSR1 LOH1 in the WT strain did not recap the high MIC50 of P4.5, is it possible that genomic variants other than Chr4 CNV and LOH1, not just other genes located in the Chr4 CNV, contribute to the FLC resistance?

We agree that there are likely factors other than the overexpression of *NCP1* and LOH at *KSR1* that contribute to the final MIC of the evolved lineage. One possibility is that a feature in addition to *NCP1* located in the Chr4 CNV or the accompanying LOH on Chr4 could impact susceptibility. This is now noted in the text (lines 384-386, 856-858).

5. Regarding NCP1, the authors showed the change of its expression level with the tet system, with or without FLC. I wonder what is the actual expression level of NCP1 in the evolved isolates (such as P3.3 and others) with Chr4 CNV, with or without FLC? Is it comparable to that of the NCP1 overexpression strain?

We thank the reviewer for this question, We added new data showing that strain P3.3, which contains the Chr4 CNV, overexpresses *NCP1* ~8-fold in the presence of fluconazole, which matches quite closely the level of overexpression of the tetO system, which we also measured to be ~8-fold upregulated using RT-qPCR (**S4 Fig C**).

6. In Fig.3B/3D, the progenitor strain (black) grew quite robustly in the presence of high concentration (256ug/ml) of FLC, which did not happen in other experiments in this study. I'm curious what happened to the progenitor strain here, and how frequent this may happen to WT SC5314?

The differences in the progenitor strain growth from 24-48 hours in Figure 3 and other panels are due to differences in tolerance. We find that tolerance is sensitive to

environmental factors, and the fact that these assays were conducted in different laboratories using different drug lots might explain these differences in tolerance. In addition, the progenitor strains for the three evolved strains shown in panel B and that of the first evolved lineage represent different versions of the SC5314 isolate and may not be completely identical.

7. In Fig. 2, all the label “KSR1 LOH” should be “KSR1 LOH1”, according to the description in the result (line 315). In Fig. 2 panel A and Fig. S8, it is hard to match the acronyms of sphingolipid species to those full names in the sphingolipid biosynthesis pathway (Fig. S1). In fact, it will help the audience to understand the data of lipid assay in Fig.2A, if the sphingolipid biosynthesis pathway is next to it.

These changes were made.

8. In the methods session, the authors stated that the filamentation assays were done in YPD broth at 37 Celsius while shaking at 200rpm. Such conditions may promote filamentation to the level shown in Fig. S9B but are less likely to induce the strong hyphae in Fig. S2B. Please confirm the method of filamentation assay in this experiment.

Both filamentation assays were performed under the same conditions. The variability may be due to the assays being performed in different laboratories. We repeated this assay and included new microscopy images in Fig. S2B to further validate these findings.

9. Again in Fig. S2B, the scale bar (5mm) looks not correct. The size of C. albicans yeast cell cannot be that large.

We thank the reviewer for pointing this out; this was an error. The scale bar for the new microscopy images in Fig. S2B is now correct.

Reviewer #2: 1. Line 137 “result in a final MIC of >256ug/mL” this should be framed in context of the progenitor strain (ex “results in a final MIC 500 fold higher than the progenitor strain”)

We made this change.

2. Line 390 figure is mislabeled – I think the authors are referencing 1G (p4.4 in 0ug/mL FLC) not 2B

We appreciate this catch. This section from the manuscript has been removed in response to other reviewer comments.

3. Line 336 the authors refer to two outliers being removed. It should be stated in the methods what tests/criteria were used to remove these two samples, as there are other samples within the same conditions that look quite different from the others (KSR1 LOH 10ug/mL FLC; 2 samples have similar profiles and one is pretty different)

We added an explanation of the criteria used to remove outliers in the methods section (lines 1268-1269 now reads: “ Two samples that clustered away from all other samples with a cluster dendrogram height of >8 were removed as outliers.”) The cluster dendrogram is shown below, with outlier samples 7 and 15:

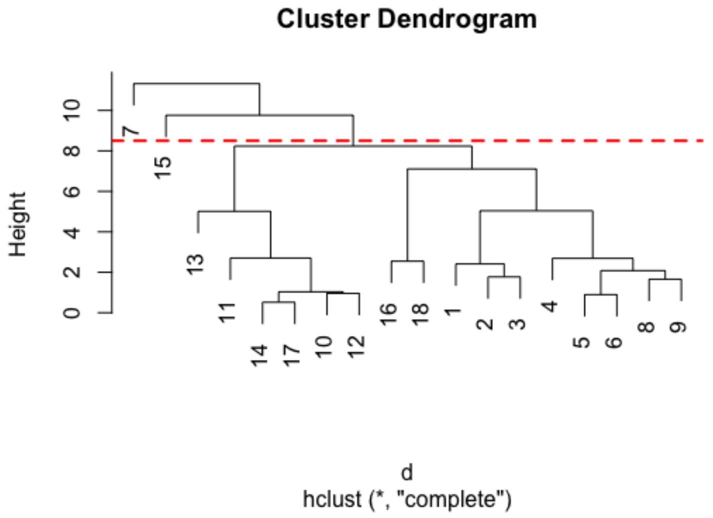

Reviewer #3: (No Response)
